# Flow cytometry, a powerful novel tool to rapidly assess bacterial viability in metal working fluids: Proof-of-principle

**DOI:** 10.1371/journal.pone.0211583

**Published:** 2019-02-01

**Authors:** Donna Vanhauteghem, Kris Audenaert, Kristel Demeyere, Fred Hoogendoorn, Geert P. J. Janssens, Evelyne Meyer

**Affiliations:** 1 Department of Nutrition, Genetics and Ethology, Faculty of Veterinary Medicine, Ghent University, Merelbeke, Belgium; 2 Laboratory of Biochemistry, Department of Pharmacology, Toxicology and Biochemistry, Faculty of Veterinary Medicine, Ghent University, Merelbeke, Belgium; 3 Department of Applied Bioscience Engineering, Faculty of Bioscience Engineering, Ghent University, Valentin Vaerwyckweg 1, Ghent, Belgium; 4 Quaker Chemical B.V., Industrieweg 7, Uithoorn, The Netherlands; University of Manitoba, CANADA

## Abstract

Metalworking fluids (MWF) are water- or oil-based liquids to cool and lubricate tools, work pieces and machines, inhibit corrosion and remove swarf. One of the major problems in the MWF industry is bacterial growth as bacterial enzymes can cause MWF degradation. In addition, bacteria can form biofilms which hamper the functioning of machines. Last but not least, some bacterial by-products are toxic (e.g. endotoxins) and present potential health risks for metalworking machine operators via the formation of aerosols. Therefore, a novel fast yet accurate analytical method to evaluate and predict the antibacterial capacity of MWF would be an important asset. As such a tool is currently lacking, the present study aimed to develop a protocol based on flow cytometry (FCM) to assess the antibacterial potential of newly developed MWF independent of bacterial growth. Results of this novel method were compared to a biochallenge test currently used in MWF industry and also to traditional plate counts. Our results represent a proof-of-principle that FCM can reliably predict the antibacterial capacity of MWF already within one day of incubation with *Escherichia coli*, *Klebsiella pneumoniae*, *Pseudomonas aeruginosa and Proteus mirabilis*, being substantially faster than the current growth-based methods.

## Introduction

The metalworking industry utilizes recirculating metalworking fluids (MWF) to cool, remove metal fines, lubricate and prevent corrosion during metal grinding and cutting procedures [1]. Metalworking fluids are complex mixtures of oils, biocides, dissolved metals, antifoaming agents and many other organic and inorganic components [2,3]. These are formulated to improve longevity of equipment, but their formulation also makes them highly prone to physical, chemical and especially microbial contamination [4,5]. Uncontrolled microbial growth significantly impacts both the MWF performance and the health of the machine operators [3,6,7]. Indeed, the resulting microbial degradation of MWF can cause corrosion of machines, tools and work pieces, as well as loss of lubricity and fluid stability, and decrease of the fluid pH due to organic acid production. Such contamination also often results in biofilm formation (visible as slime which can plug filters) and in unacceptable odors, but also carries important health risks for operators [8,9]. On the other hand, knowledge on the MWF deterioration capacity of microorganisms may also provide an important solution towards a safe and economical disposal of operationally exhausted MWF [10,11,12].

Microbial contamination of MWF is inherently related to the composition of these complex fluids. More than three decades ago, Foxall-Van Aken et al. [13] already stated that 3 important classes of compounds are usually present in MWF at a sufficient concentration to support microbial growth, being oil, petroleum sulphonates and fatty acids. In addition to these key carbon sources, breakdown products of other microorganisms may also serve as nutrients [4]. Nevertheless, the highly selective nature of those substrates limits the microbial diversity in MWF [10,14,15]. Consequently, it is the general chemistry of a MWF, rather than any particular component hereof, which selects for the microbial community composition [15]. Many studies have identified Pseudomonads, including *Pseudomonas aeruginosa*, as the predominant species [3,10,14,16,17,18]. A variety of other bacterial classes, including *Klebsiella pneumoniae*, *Proteus mirabilis* and *Escherichia coli* has been described to also frequently contaminate MWF [4,9,17,18,19,20].

Currently, the use of biocides is the most common strategy for the control of microbial growth in MWF, with formaldehyde condensates being the most popular chemical agents [6,21]. However, the use (of combinations) of biocides is subjected to severe regulations and limitations. Consequently, there is an ongoing search for novel MWF biocides that may provide superior alternatives. For this purpose, it is of critical importance to evaluate each of such candidate biocide against appropriate problem strains, both in controlled and in used MWF matrix conditions [22]. Regarding the former, the individual potential of candidate biocidal compounds should be verified in such a controlled environmental set-up as an initial screening.

Yet, research in this specific field is hampered by the fact that the conventional culture-based methods used for the evaluation of the biocidal potential of MWF lack both speed and specificity. Most importantly, the stressed and/or non-culturable fraction of this bacterial population, which equally contributes to the described detrimental effects, remains undetected in culture-based analysis [23]. This has led to an increasing interest in developing alternative methods that can provide real-time information on the microbial viability in MWF. Several studies have been performed with a variation of bacterial species including PCR [23,24], FISH [25], ATP quantification [26], and also flow cytometry (FCM) [27,28]. Both latter studies from the same group illustrated the problems occurring due to interference of the MWF matrix with FCM and highlight the need to isolate the micro-organisms from this matrix prior to such analysis.

In the present study, an isolation method of selected Gram-negative bacterial species all relevant in the context of MWF is at first described complementary to our recent report on fungal contamination of MWF [29]. Secondly, this optimized sample preparation was the basis for the subsequent FCM evaluation of real-time bacterial viability using carefully optimised conditions. Thirdly, for comparative purposes, plate counts were performed and our novel FCM method was further validated and its predictive value on the biocidal potential of the screened MWF confirmed, by comparing these data with the time consuming American Society for Testing and Materials (ASTM E2275) method traditionally used in MWF industry.

## Materials and methods

### Test compounds

#### Quakercool 3530 FF (product 270)

This product is described as a multi-purpose MWF used mainly for ferrous alloys. It contains polyborates which results in an excellent pH buffering capacity. As a lubricant it consists of a medium amount of mineral oils and a low amount of synthetic esters. It does not contain any biocides.

#### Quakercool 7110 BF (product 258)

This high-end product is mainly used for difficult machining operations on aluminium alloys. It is boron-free and relies on a combination of primary and tertiary alkanolamines, in combination with a proprietary, innovative boron replacement for pH buffering. For lubrication it consists of a high level of mineral oils and a medium amount of synthetic esters. It does not contain any biocides.

#### Quakercool 7601 BFFR (product 869)

This high-end product is mainly used for machining aluminium, titanium and nickel alloys, specifically in aerospace applications. As lubricant it contains a low amount of mineral oils and a high amount of different synthetic esters, combined with a phosphorous based anti-wear agent. It is boron-free, and contains an isothiazolone based biocide. The special blend of specific amines not only inhibits the growth of gram negative bacteria, but also of mycobacteria and fungi.

#### Experimental Product 284 (prototype 0018.2.4)

This product is an experimental product which was not further developed due to insufficient resistance against microbiological growth. It contains a medium amount of mineral oil and synthetic esters, a low amount of primary and tertiary alkanolamines. It is boron-free and contains no biocides.

### Preparation of the emulsions

All emulsions were first prepared at a concentration of 6% end use dilution in Conshohocken tap water. The pH of the emulsions was equilibrated by bubbling through air for 24 hours (h), at an air flow rate of 350 ml/min. The uptake of carbon dioxide from the air brings down the pH to an equilibrium value. pH after aeration was recorded as the starting pH for the biochallenge test. Samples that passed the 8 week challenge at 6%, were further diluted to 4%.

Starting pH values for the biochallenge test were 9.15, 8.93, 9.19 and 9.27 for P270, P284, P258 and P869 respectively. For the flow cytometric and plate counts analyses separate series of the emulsions were prepared with pH 9.00 and 9.50.

### Bacterial strains and culture

*Escherichia coli* (ATCC 8739), *Pseudomonas aeruginosa* (ATCC 27853) and *Klebsiella pneumoniae* (ATCC 13883) were obtained from the DSMZ culture collection (Germany). *Proteus mirabilis* (ATCC 7002) was obtained from LGC Standards. These bacterial species were selected based on ASTM E686-91 and ASTM E2275-03.

For flow cytometric analysis and plate counts, *E*. *coli*, *P*. *aeruginosa*, *K*. *pneumoniae and P*. *mirabilis* were separately grown overnight at 37°C in Müller-Hinton broth (MH, Oxoid Limited, Hampshire, United Kingdom). The bacteria were then collected by centrifugation (10 000 x g, 2 min, room temperature) of 1 ml of bacterial culture and then resuspended in 1 ml of sterile PBS.

### Exposure conditions for flow cytometry and plate counts

The four MWF were inoculated with the separate bacterial cultures to a final concentration of 4.5*10^6^–1*10^7^ CFU/ml MWF, without affecting the final MWF concentration. As a control sample, sterile 0.9% NaCl (pH 7.4) was also inoculated with the same bacterial concentrations. The inoculated samples were then incubated at 28°C and samples were taken at different time points (0, 5 and 24h, and after 1 week) for further analysis.

### Flow cytometric viability measurement

#### Extraction of bacteria from the MWF

As MWF are complex matrices comprising different types of mineral oils, amines and synthetic esters amongst others, appropriate sample preparation was necessary to allow FCM assessment of the bacteria present in these MWF. Optimization of the isolation process was done for all four types of MWF used to achieve a common extraction procedure for these MWF matrices. Incubation in the MWF resulted in residual pollution of the bacterial population with MWF components after a 1-step isolation of the bacteria by centrifugation. This pollution was attributed to the oil vesicles present in the MWF matrix. Therefore, the bacteria were washed three times with ice-cooled sterile saline in Eppendorf tubes (4000g, 10 min, 4°C) to remove the matrix pollution. After the last wash step, the pellet was resuspended in 20 μl sterile saline and transferred to a polystyrene round base flow cytometry tube.

#### Fluorescence staining and viability assessment of the extracted bacteria

All data were obtained using a FACSCanto flow cytometer (Becton, Dickinson and Company, Erembodegem, Belgium), and acquired and processed using FacsDiva software (Becton, Dickinson and Company, Franklin Lakes, NJ, USA). All experiments were performed in triplicate.

Bacterial viability was assessed using the LIVE/DEAD BacLight^TM^ kit (Molecular Probes Eugene, OR, USA) as described by the manufacturer. This bacterial viability kit is widely used in flow cytometry and consists of two nucleic acid stains: green fluorescent SYTO 9 is cell-permeable and freely enters all tested bacteria, either live or dead, while red fluorescent propidium iodide (PI) can only enter membrane-comprised cells [30]. In our set-up, 977 μl of sterile saline cell suspension was added to 20 μl of the treated bacteria. These samples were immediately stained with 3 μl of a mixture of SYTO 9 (5 μM final concentration) and PI (30 μM final concentration) and incubated for 15 minutes in the dark at room temperature. FCM measurements were performed immediately thereafter.

### Plate counts

The number of CFU per ml was assessed by conventional plate count, which is based on CFU values obtained from a 10-fold serial dilution of each sample plated on Tryptone Soy Agar (Oxoid Limited, Hampshire, United Kingdom) and incubated overnight at 37°C. These plate counts determine the number of culturable bacteria in each sample. All data are the result of triplicate experiments.

### Biochallenge test

The biological resistance of the fluids was assessed in a test essentially equivalent to the method described in ASTM E686-91, Standard Method for Evaluation of Antimicrobial Agents in Aqueous Metalworking Fluids, with the following adaptations. All fluids were initially inoculated with 10% of freshly prepared, mixed inoculum in Trypticase Soy Broth, consisting of *E*. *coli*, *K*. *pneumoniae*, *P*. *aeruginosa*, *P*. *mirabilis*. In the consecutive weeks inoculations were done with 2% of freshly prepared inoculum of the same bacterial composition. The bacteria in the mixed inoculi were enumerated by plate count on Standard Plate Count Agar (DF 0479173).

Prior to the weekly additions of inoculum, the emulsions were sampled and the number of surviving bacteria were enumerated by plate count on Standard Plate Count Agar (DF 0479173).

The test was run for 8 consecutive cycles (1 cycle = 1 week), or until failure. Failure is defined as the presence of >5 log CFU/ml for 2 consecutive weeks. Emulsions that passed eight cycles were further diluted down to 4% and the test was continued for an additional 8 weeks or until failure.

### Statistical analysis

A completely randomized block design (general linear model) was used. To assess the effect of the different treatments, a Tukey test was performed per time point and per bacterial species with a reliability of α = 0.05. The Tukey algorithm was applied to compensate for the increasing type I error during multiple pairwise comparisons of treatment.

## Results

### Flow cytometric viability measurement

**Fig 1:**
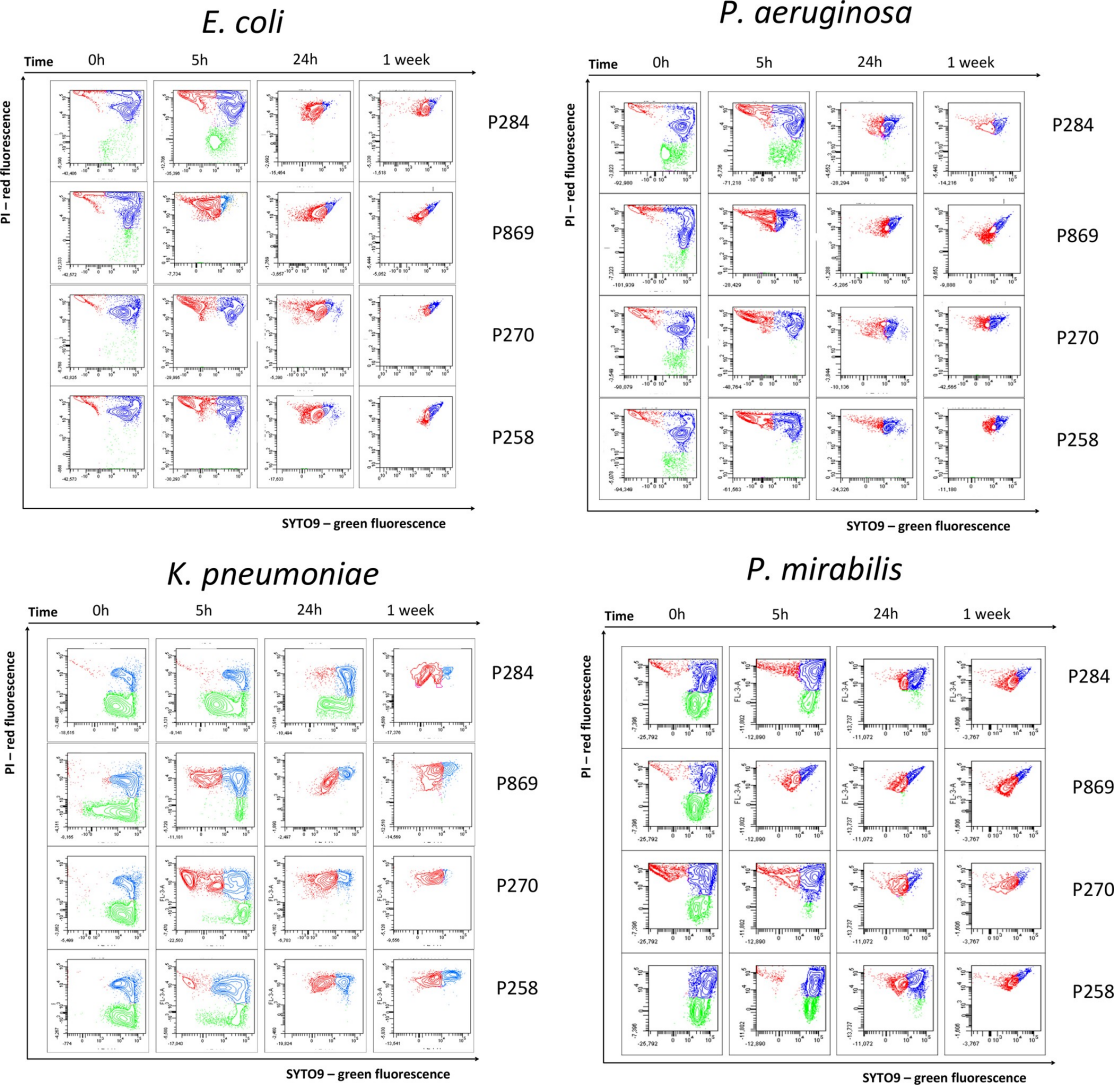
Flow cytometric SYTO 9/PI dual staining contour plots. Flow cytometric SYTO 9/PI dual staining contour plots presenting *E*. *coli*, *P*. *aeruginosa*, *K*. *pneumoniae* and *P*. *mirabilis* viability at different incubation times with each of the 4 MWF (P284, P869, P270, P258) at pH 9.0 and compared to PBS (negative control). The green region corresponds to the subpopulation of viable cells with an intact plasma membrane, the blue region corresponds to the subpopulation of intermediate cells with (partly) damaged membranes and the red region corresponds to the subpopulation of dead cells with irreversibly damaged membranes.

As previously shown for *E*. *coli* [31], flow cytometric analysis of membrane integrity with SYTO 9/PI dual staining revealed a unique fluorescence pattern directly related to the degree of membrane damage. Our optimized FCM protocol was now used to monitor the viability of *E*. *coli*, *P*. *aeruginosa*, *K*. *pneumoniae* and *P*. *mirabilis*. in 4 different MWF and each at 2 different pH values (pH 9.0 and 9.5). A negative control sample (sterile PBS) was included in all experiments for comparison purposes. Three bacterial subpopulations were thus identified: membrane-intact live bacteria, membrane-damaged “intermediates” and dead bacteria. The FCM contour plots obtained at pH 9.0 are shown in Fig 1, those of the incubations at pH 9.5 are shown in S1 Fig.

#### Influence of short term incubation (i.e. 5h and 24h)

Our FCM data show that the viability of *K*. *pneumoniae*, *P*. *aeruginosa* and *P*. *mirabilis* was the least affected upon their exposure to MWF P284, in contrast to *E*.*coli* at 24h of exposure. The antibacterial influence of the other 3 MWF was even more species-dependent (Fig 1). The increase in percentage of dead bacteria between the start of the experiment (t0) and after 24h of incubation is summarized for each of the 4 MWF and separate bacterial species (Table 1). The antibacterial influence of the MWF was species-dependent (Fig 1).

**Table 1:** Increase in the percentage of dead bacteria for each of the 4 bacterial species between t0 and t24 after exposure to each of the 4 MWF at pH 9.0.

Increase in % of dead bacteria after 24h incubation				
	P284	P869	P270	P258
***E*. *coli***	79.8	73.8	68.9	63.8
***P*. *aeruginosa***	8.4	4.1	4.6	-1.8
***K*. *pneumoniae***	3.4	59.2	78.7	74.3
***P*. *mirabilis***	19.7	34.0	42.7	35.3

Increase in the percentage of the dead bacterial subpopulation of *E*. *coli*, *P*. *aeruginosa*, *K*. *pneumoniae* and *P*. *mirabilis* between the start of the incubation (t0) and after 1 day (t24).

The difference in susceptibility to the 4 MWF comparing *E*. *coli*, *K*. *pneumoniae*, *P*. *aeruginosa* and *P*. *mirabilis*, at both incubation times (t0, t24) and both pH values (pH 9.0 and 9.5) are presented in Table 2, showing the percentages of the three bacterial subpopulations for all incubation conditions.

**Table 2:** Overview of the percentages of the bacterial subpopulations.

			Incubation time																							
bacterial spp.	pH	treatment	0h						5h						24h						1 week					
			L		I		D		L		I		D		L		I		D		L		I		D	
*E*. *coli*	9	P284	3.4	**a**	88.1	**bc**	8.5	**a**	11.6	**a**	62.8	**b**	25.5	**ab**	0.1	**a**	11.8	**a**	88.3	**c**	0	**a**	40.7	**a**	59.2	**c**
		P869	3.3	**a**	83.4	**b**	12.5	**a**	0.4	**a**	8.3	**a**	92.7	**b**	0.2	**a**	41.5	**b**	56.3	**b**	0	**a**	53.3	**a**	47.1	**bc**
		P270	1.2	**a**	91.1	**c**	7.7	**a**	0.2	**a**	47.7	**ab**	52.2	**c**	0.1	**a**	28.3	**ab**	76.6	**c**	0	**a**	66.9	**b**	33.1	**ab**
		P258	1	**a**	90.1	**c**	8.8	**a**	0.7	**a**	66.5	**ab**	33	**ab**	0.2	**a**	30.6	**ab**	72.6	**bc**	0	**a**	75.5	**b**	24.5	**a**
* *	9.5	P284	0.6		86.8		12.5		1		60.1		39.2		0.2		22.9		82.3		0		39.2		60.6	
		P869	1		86		12.6		0.2		18.8		84.4		0		45.7		60.7		0		49.5		50.5	
		P270	0.5		94		5.5		0.1		41.6		58.3		0.1		18.8		83.7		0		67		33	
		P258	0.6		93.8		4.8		0.4		40.8		59		0.2		34.9		69.2		0		65.2		36.6	
		neg con	44.4	**b**	23.7	**a**	31.9	**b**	60	**b**	28.7	**ab**	11.6	**a**	64.5	**b**	28.9	**ab**	6.6	**a**	37.9	**b**	39	**a**	23.5	**a**
*P*. *aeruginosa*	9	P284	10.7	**a**	83.7	**b**	5.5	**a**	9.8	**a**	61.3	**a**	28.9	**a**	0.2	**a**	87.7	**b**	13.9	**cd**	0.1	**a**	76.1	**b**	23.8	**b**
		P869	2.7	**a**	89.3	**b**	8.1	**b**	0.2	**a**	36.3	**a**	62	**a**	0.7	**a**	87.7	**b**	12.2	**bc**	0.3	**a**	79.9	**b**	19.2	**b**
		P270	4.3	**a**	83.7	**b**	12	**c**	0.2	**a**	41.1	**a**	58.7	**a**	0	**a**	84.4	**b**	16.6	**d**	0.4	**a**	84.2	**b**	15.4	**ab**
		P258	4.3	**a**	87.9	**b**	7.9	**b**	0.9	**a**	54.4	**a**	44.8	**a**	0.1	**a**	93.8	**c**	6.1	**a**	0.4	**a**	81.7	**b**	18	**ab**
* *	9.5	P284	2.3		92		5.7		2.4		65.4		32.2		0.2		84		15.3		0.1		78.6		21.4	
		P869	0.6		90.3		9.1		0		70.2		27.3		0.4		87.2		12.4		0.2		79		20.8	
		P270	0.7		87.1		12.3		1		39.3		59.6		0.4		81.7		18		0		77		23	
		P258	3.8		88.3		8		0.3		45.9		54		0.3		94.1		5.6		0.6		81.8		17.5	
		neg con	60.3	**b**	23.7	**a**	16	**d**	74.4	**b**	17.9	**a**	7.5	**a**	68.6	**b**	20.2	**a**	10.1	**b**	76.2	**b**	15.9	**a**	7.5	**a**
*K*. *pneumoniae*	9	P284	77.2	**b**	22.2	**ab**	0.7	**a**	69.8	**b**	28.6	**ab**	1.2	**a**	44.6	**a**	51.3	**b**	4.1	**ab**	0	**a**	17.7	**a**	79.9	**b**
		P869	52	**a**	45.5	**bc**	2.1	**b**	11.5	**a**	56.9	**ab**	32	**a**	0.1	**a**	36.2	**ab**	61.3	**ab**	0.2	**a**	35.5	**a**	65.2	**b**
		P270	72.7	**ab**	26.6	**abc**	0.6	**a**	14.5	**a**	37.5	**ab**	48.2	**a**	0.1	**a**	22.1	**ab**	79.3	**ab**	0	**a**	8.2	**a**	92	**b**
		P258	51.1	**a**	48.7	**c**	0.4	**a**	12.2	**a**	82.7	**b**	5.1	**a**	0.1	**a**	25.4	**ab**	74.7	**b**	0	**a**	45.7	**a**	54.7	**b**
* *	9.5	P284	66.2		33.1		0.7		52.5		41.1		6.5		0.2		54.1		45.9		0.2		17.5		79.4	
		P869	28.8		69.5		1.8		0.3		56.2		43.7		0.1		66.4		34.3		0		41.6		58.9	
		P270	66		33.6		0.5		29.4		33.1		37.6		0.9		24.2		74.7		0		6.8		93.3	
		P258	29.3		70.3		0.6		7.9		50.1		42.2		0		11.6		88.6		0		15.5		84.9	
		neg con	98.3	**b**	1.5	**abc**	0.2	**a**	95	**b**	4.4	**a**	0.6	**a**	96.9	**b**	2	**a**	1.1	**a**	90.4	**b**	5.7	**a**	3.7	**a**
*P*. *mirabilis*	9	P284	47.4	**ab**	48.9	**b**	3.9	**a**	16.2	**a**	75	**a**	9.1	**a**	1.5	**a**	75	**b**	23.6	**ab**	0	**a**	46.3	**a**	53.7	**b**
		P869	64.1	**ab**	33.8	**b**	2.4	**a**	0.1	**a**	64.8	**a**	36.5	**a**	0.5	**a**	63.2	**ab**	36.4	**b**	0	**a**	34.6	**a**	65.4	**bc**
		P270	22.6	**a**	53.7	**b**	23.9	**a**	1.4	**a**	74.7	**a**	24.3	**a**	0.7	**a**	32.6	**ab**	66.6	**b**	0	**a**	24.2	**a**	75.8	**c**
		P258	52	**a**	48.4	**b**	0.1	**a**	40.6	**a**	57.1	**a**	2.6	**a**	1.2	**a**	62	**ab**	35.4	**ab**	0	**a**	73.1	**a**	54.3	**b**
* *	9.5	P284	46.8		50.6		3		19.5		73.2		7.3		0.2		67.5		32.4		0		42.2		57.8	
		P869	33.6		61.3		5.3		0.2		26.1		74.6		0.5		26.7		73		0		37		63	
		P270	26.7		62.6		10.8		0.1		76		24.3		0.1		30.9		67.5		0		32.9		73.1	
		P258	37.4		57.9		5		2.3		76.9		21.2		0.4		49.3		48.3		0		40.2		59.8	
		neg con	91.4	**b**	5.8	**a**	2.8	**a**	79.4	**b**	19.2	**a**	1.6	**a**	85.4	**b**	14.2	**a**	0.7	**a**	58	**b**	35.8	**a**	5.5	**a**

Overview of the percentages of the live (L), intermediate (I) and dead (D) bacterial subpopulations based on their membrane integrity evaluation. Different letters indicate statistical differences (p<0.05).

For *E*. *coli* at pH 9.0 and after 5h of incubation, the highest percentage of dead bacteria (92.7% cell death) was seen with P869. After 24h of incubation, the highest percentage of dead bacteria was seen with P270 (76.6% cell death), closely followed by P258 (72.6% cell death). Results at pH 9.5 were comparable (S1 Fig). For *P*. *aeruginosa* at pH 9.0 and after 5h of incubation, the highest percentage of dead bacteria was also seen with P869 (62.0% cell death), closely followed by P270 (58.7% cell death). After 24h of incubation, the highest percentage of dead bacteria was seen with P270 (16.6% cell death). For *P*. *aeruginosa* results also show that after 24h of incubation the intermediate bacteria are the predominant viability state present (i.e 87.7%, 87.7%, 84.4% and 93.8% of intermediate bacteria with P284, P869, P270 and P258 respectively). Results at pH 9.5 were comparable to pH 9.0 (S1 Fig). For *K*. *pneumoniae* at pH 9.0 and after 5h of incubation the highest percentage of dead was seen with P270 (48.2% cell death), followed by P869 (32.0% cell death). After 24h of incubation the highest percentage of dead bacteria was seen with P270 (79.3% cell death), closely followed by P258 (74.7% cell death) and P869 (61.3% cell death). Results at pH 9.5 were comparable for P869 (43.7% cell death), P270 (37.6% cell death) and P258 (42.2% cell death) (S1 Fig). For *P*. *mirabilis* at pH 9.0 and after 5h incubation the highest percentage of dead was seen with P869 (36.5% cell death), closely followed by P270 (24.3% cell death). After 24h of incubation, P270 has the highest percentage of dead bacteria (66.6% cell death). Results at pH 9.5 show the highest percentage of dead bacteria with P869 (74.6%) after 5h of incubation, whereas at 24h of incubation results for both P869 and P270 were comparable (73.0% and 67.5%, respectively) (S1 Fig).

#### Influence of long term incubation (i.e. 1 week)

Remarkably, for *E*. *coli*, at pH 9.0 and after 1 week of incubation the percentage of dead bacteria for all MWF (59.2%, 47.1%, 33.1% and 24.5% for P284, P869, P270 and P258, respectively) was lower than after 24h of incubation (88.3%, 56.3%, 76.6% and 72.6% for P284, P869, P270 and P258, respectively). Consequently the percentage of intermediate bacteria was systematically higher after 1 week of incubation (Table 2). For *P*. *aeruginosa* this overall effect was already observed after 24h of incubation and persists as the bacterial population consisted mainly of intermediate bacteria after 1 week of incubation during at pH 9.0 (76.1%, 79.9%, 84.2% and 81.7% of intermediate bacteria with P284, P869, P270 and P258, respectively). In marked contrast to both *E*.*coli* and *P*. *aeruginosa*, for *K*. *pneumoniae* the percentage of dead bacteria increased after 1w of incubation at pH 9.0 (79.9%, 65.2%, 92.0% and 54.7% for P284, P869, P270 and P258, respectively) compared to 24h after incubation with P284 (4.1% cell death), P869 (61.3% cell death) and P270 (79.3% cell death) but not with P258 (74.7% cell death). For *P*. *mirabilis* after 1 w of incubation at pH 9.0 (53.7%, 65.4%, 75.8% and 54.3% for P284, P869, P270 and P258, respectively) a higher percentage of dead bacteria was seen compared to after 24h of incubation (23.6%, 36.4%, 66.6% and 35.4% for P284, P869, P270 and P258, respectively).

Results of pH 9.5 are comparable to the effects seen at pH 9.0 (S1 Fig).

### Plate counts

**Fig 2:**
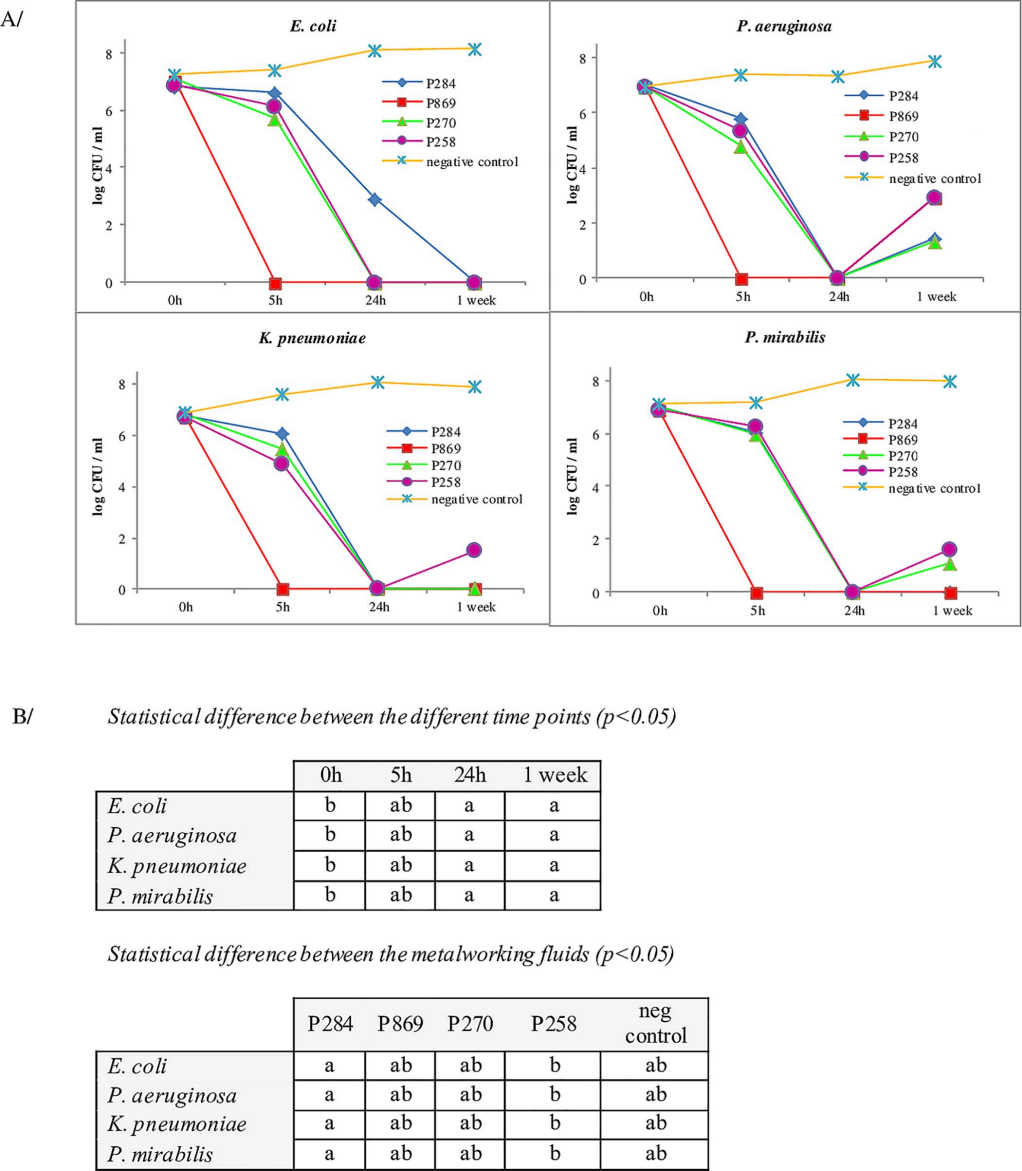
Plate counts and statistical analysis. (A) Plate counts of *E*. *coli*, *P*. *aeruginosa*, *K*. *pneumoniae* and *P*. *mirabilis* representing bacterial culturability. Bacterial populations were exposed for different incubation times to either P284, P869, P270, P258 at pH 9.5 and compared to PBS (negative control). (B) Different letters indicate statistical differences (p<0.05).

Complementary to the flow cytometric evaluation of bacterial viability, the bacterial culturability was assessed through traditional plate counts for in-house comparative purposes. Mean values of *E*. *coli*, *K*. *pneumoniae*, *P*. *aeruginosa and P*. *mirabilis* incubated with the 4 different MWF and a negative control (0.9% NaCl) are presented in Fig 2. Culturability of all 4 bacterial species was most affected by exposure to P869 after 5h and longer incubation times. For *E*. *coli*, P284 had the lowest antibacterial effect (still 2.92 log CFU/ml after 24h of incubation), while for the 3 other bacterial species P284 had a similar effect as P270 and P258, i.e. after 24h of incubation at pH 9.0, no bacterial growth was observed. Remarkably, after 1 week of incubation at pH 9.0 bacterial growth was seen for *P*. *aeruginosa* incubated with all MWF (1.44, 2.94, 1.33 and 2.95 log CFU/ml with P284, P869, P270 and P258, respectively), for *K*. *pneumoniae* when incubated with P258 (1.50 log CFU/ml) and for *P*. *mirabilis* when incubated with P258 (1.62 log CFU/ml) and P270 (1.09 log CFU/ml).

### Biochallenge test

Results of this slightly adapted Standard Method for Evaluation of Antimicrobial Agents in Aqueous Metalworking Fluids (Table 3) showed that P284 had a bacterial count >5 log CFU/ml after the first period of 8 weeks with an emulsion concentration of 6%. This product was thus not further exposed to the bacterial mix and failure was defined after 7 cycles. On the other hand, P270, P258 and P869 all showed satisfactory plate counts ≤ 2 log CFU/ml after the first 8 cycles. These 3 MWF were therefore further diluted to 4% to continue the biochallenge test. Plate counts showed that P270 failed after 3 extra cycles (i.e. failure after 11 cycles) and that P 258 failed after 2 extra cycles (i.e. failure after 10 cycles) at those lower emulsion concentrations. Only P869 was able to resist the bacterial challenge for the whole second 8 week period (i.e. failure after >16 cycles). Table 3 presents an overview of all bacterial counts during the biochallenge test. From these plate count data an overall ranking of the products could be made according to their bio-resistancy: P869 > P270 > P258 > P284, with P869 being the most resistant and P284 the least resistant to *E*. *coli*, *K*. *pneumoniae*, *P*. *aeruginosa and P*. *mirabilis*.

**Table 3:** Overview of all bacterial counts during the biochallenge test.

		Surviving bacteria (log CFU/ml)			
	MWF	P270	P284	P258	P869
**Inoculum (log CFU/ml)**	**week #**	Emulsion concentration 6%			
7.54	1	2.00	2.00	<2.00	<2.00
7.73	2	2.00	4.00	2.00	<2.00
7.64	3	3.00	4.00	2.00	<2.00
7.56	4	<2.00	3.00	<2.00	2.00
7.32	5	2.00	3.00	<2.00	<2.00
7.36	6	2.00	4.00	<2.00	<2.00
7.32	7	2.00	4.00	2.00	<2.00
7.32	8	<2.00	7.40	2.00	<2.00
		Emulsion diluted to 4%			
7.41	9	3.00	terminated	<2.00	<2.00
7.43	10	3.00		3.00	4.00
7.61	11	3.00		7.00	<2.00
7.69	12	5.00		7.00	<2.00
7.66	13	>7.00		terminated	2.00
7.69	14	terminated			<2.00
7.63	15				4.00
7.65	16				3.00
**Failure in week**		13	8	11	>16

All fluids were initially inoculated with 10% of freshly prepared, mixed inoculum of *E*. *coli*, *K*. *pneumoniae*, *P*. *aeruginosa and P*. *mirabilis* in TSB broth. In the consecutive weeks inoculations were done with 2% of freshly prepared inoculum of the same bacterial composition. Prior to the weekly additions of inoculum, the emulsions were sampled and the number of surviving bacteria were enumerated by plate count on Standard Plate Count Agar. The test was run for 8 consecutive cycles (1 cycle = 1 week), or until failure. Failure is defined as the presence of >5 log CFU/ml for 2 consecutive weeks. Emulsions that passed eight cycles were further diluted down to 4% and the test was continued for an additional 8 weeks or until failure.

## Discussion

Results show that the susceptibility to the MWF compounds is predominantly bacterial species-dependent. When the results of an incubation time up to 24h are compared, *K*. *pneumoniae* and *P*. *mirabilis* are the most resistant to the antimicrobial effects of the MWF matrices. When incubated for 1 week, *P*. *aeruginosa* and *P*. *mirabilis* appeared to be more resistant to the antimicrobial effects of the MWF. When comparing the different MWF, at 5h of incubation P869 has a greater antibacterial effect, however, after 24h of incubation exposure to P270 results in the highest percentage of dead bacteria. In general, the antibacterial effect of the MWF appears stronger at pH 9.5 compared to pH 9.0, however the observed trends of decreased bacterial viability are comparable at both pH values and there was no statistically significant effect of pH.

When relating the results more specifically to the composition of the different MWF used, several observations can be made. The MWF P270, P258 and P284 all contain no specific biocides, whereas P869 does contain an isothiazolone based biocide. Isothiazolone biocides are widely used in different water-based industrial systems [32,33] and the presence of this compound could explain the better antibacterial characteristics of P869. The antibacterial action of isothiazolone biocides is based on the disruption of metabolic pathways by a broad enzyme inhibition, resulting in the loss of viability and inhibition of growth, followed by a rapid cell death [32].

Other compounds with antibacterial effects are the boron-based compounds [34,35]. Only P270 contained polyborates which will have contributed to the antibacterial effect. Sandin et al. [2] described an effect of boric acid on the bacterial cell wall, which could explain the rapidly detected effect in the bacterial membrane by our flow cytometry protocol. The MWF P258 and P284 do not contain a biocide, nor any boron-based compounds, however alkanolamines were added to these MWF. The antibacterial potential of alkanolamines has also been described [36,37]. The MWF P284 contains a lesser amount of these alkanolamines, which could explain the poor antibacterial capacity of this MWF.

The current results show a remarkable concordance between the prediction based on the FCM data and the industrial biochallenge test ranking the 4 MWF according to their bio-resistance. Whereas up to 16 weeks were needed for the latter ranking, FCM viability staining generated the same conclusion after only 24h of incubation. Moreover, the FCM data allowed additional differentiation in the antibacterial activity of the different MWF and also on the susceptibly of the different species of bacteria by the identification of 3 separate bacterial populations, i.e. live, intermediate and dead bacteria. More specifically, the identification of a third subpopulation of “intermediate” bacteria beside the viable and dead subpopulations yields important information on the antimicrobial potential of the different MWF. Moreover, this extra population of bacteria provides potential information on the viability of the bacteria that is missed when in only culturability is taken into account.

There are only a handful of studies describing the use of FCM to evaluate microbial viability in MWF. Recently, our group has developed a novel method that allows the accurate prediction of the fungicidal potential of MWF using FCM viability measurements [29]. This method uses a simple centrifugation protocol to isolate fungal conidia from MWF. Previously, the FCM detection and quantification of mycobacteria was described by Chang et al. [27], performing the staining in the MWF matrix, which resulted in significant interferences in their viability assessment. To overcome this matrix interference, they subsequently [28] attempted to isolate the mycobacteria by immunomagnetic separation and centrifugation, but again reported suboptimal results. Chang et al. [27] also emphasizes the importance of separating bacteria from the MWF in order to measure specific characteristics of bacterial populations. In accordance with our novel protocol developed for fungal conidia, which comprises of 2 straightforward washing steps, we were able to isolate *E*. *coli*, *K*. *pneumoniae*, *P*. *aeruginosa and P*. *mirabilis* from MWF combining centrifugation and several wash steps at 4°C. This simple sample preparation elegantly avoided matrix interference with our bacterial viability assessment after incubation with different MWF matrices. The strength of this isolation method lies in its simplicity and it can be performed in MWF developing laboratories. Also, the use of FCM has become a standard method in several scientific fields, which has led the development of affordable user-friendly flow cytometers. Most importantly, there is a very significant time gain in evaluating the antibacterial potential of newly developed MWF using our novel FCM method (24h) compared to the traditionally used biochallenge method (8 weeks).

Traditional enumeration methods for bacteria in MWF include both direct and indirect detection. Typically, dip slides used to grow and subsequently count the number of bacteria present in MWF are very laborious and time consuming. Moreover, they underestimate the actual distribution of the bacteria present in the MWF [18]. The past decade, several novel methods have been described that potentially meet the industry’s need for a test method that can rapidly estimate the total number of bacteria, such as the ATP bioluminescence assay [38,39] and various adapted microscopy-based direct count methods [25,40,41]. More recently, substantial efforts have also been made to detect and quantify specific microbial types in MWF without the need for their culture [42]. Nevertheless, none of these alternatives have been widely accepted. In the current study, a straightforward novel growth-independent protocol that allows the reliable prediction of the antibacterial potential of experimentally contaminated MWF within only 24h, is presented and applied in parallel to the ASTM standard method used in the MWF industry. The latter method is growth-based and requires several weeks of incubation. Therefore, our protocol represents a major saving of both time and budget in the development of novel MWF formulations. This is convincingly demonstrated in our proof-of-principle concept on the highly predictive potential of our flow cytometry based protocol for the evaluation of antibacterial capacity of 4 MWF against *E*. *coli*, *K*. *pneumoniae*, *P*. *aeruginosa and P*. *mirabilis*. Of relevance, such predictive power was recently also described by our group for the antifungal capacity, after incubation of different commercial MWF with *Fusarium solani* [29].

Generally, the use of flow cytometry in combination with specific fluorescent stains provides detailed information and different states of the bacterial viability whereas traditional techniques like agar plating and microscopy only provide limited information [25]. Moreover, recently novel flow cytometer models of (e.g. the Cytoflex system of Beckman Coulter) have been developed with a very high sensitivity using adopted strategies for single molecule fluorescence detection in sheathed flow. Using label-free side scatter detection, a single bacterial cell can be well discriminated from the instrumentation background and total bacterial counts can be made based on a volumetric control that enable a direct quantification of the bacterial concentration [43,44].

The microscopy-based counting reveals the total population of cells and plate counting only yields the culturable fraction of the population. A major discrepancy between those different types of counts is often reported. The LIVE/DEAD BacLight^TM^ dual staining used in the current study has been described for viability analysis of bacterial species in several biological matrices such as seawater, drinking water, soil and food products [45,46,47,48]. Only in a few studies the use of this staining has been reported in MWF for either Mycobacteria [25] or by our group for fungal conidia [29]. Nonetheless, the ability of the SYTO 9/PI staining to differentiate different viability states of bacteria within a bacterial population has been widely described including also reports from our group [30,31,49].

When comparing the traditional biochallenge technique and the FCM method, it is obvious that the FCM technique is faster, less labor-intensive and provides more detailed information about the antibacterial potential of MWF. For these reasons, the here developed FCM method is strongly suggested to be valuable in predicting the antibacterial capacity of new MWF formulations. Therefore, our straightforward novel protocol might be implemented in an industrial setting to evaluate the antibacterial properties of new MWF formulations in a considerably more time- and cost-efficient manner.

## Conclusion

This paper provides novel data on the predictive power of FCM in assessing the antibacterial capacity of MWF formulations. The development of an accurate analytical tool to evaluate bacterial viability in MWF is an asset important step to tackle this problem. In the present study we developed a FCM method to measure bacterial viability, based on membrane integrity, in MWF using *Escherichia coli*, *Pseudomonas aeruginosa*, *Klebsiella pneumoniae* and *Proteus mirabilis* as model organisms. We compared our novel flow cytometric method with a biochallenge test currently used in MWF industry and also with traditional plate counts. This study is a proof-of-principle which is timely as it meets the current increasing needs of industrial stakeholders and the academic world to extend traditional microbiological test methods with novel high-throughput consensus methods which can later be adopted by industrial stakeholders. The combination of bacterial isolation, fluorescent staining and FCM analysis holds large potential to enhance and greatly accelerate the quantitative and qualitative evaluation of bacterial viability in MWF.

## Supporting information

S1 Fig Flow cytometric SYTO 9/PI dual staining contour plots presenting the *E*. *coli*, *P*. *aeruginosa*, *K*. *pneumoniae* and *P*. *mirabilis* viability at different incubations with each of the 4 MWF (P284, P869, P270, P258) at pH 9.5 compared to PBS (negative control). The green region corresponds to the subpopulation of viable cells with an intact plasma membrane, the blue region corresponds to the subpopulation of intermediate cells.
(PPTX)
