# Supplementary material for: Flow cytometry, a powerful novel tool to rapidly assess bacterial viability in metal working fluids: Proof-of-principle
Source: PLoS One. 2019 Feb 1;14(2):e0211583. doi: 10.1371/journal.pone.0211583 (PMC6358156; doi:10.1371/journal.pone.0211583)
Supplement: S1 Fig — The green region corresponds to the subpopulation of viable cells with an intact plasma membrane, the blue region corresponds to the subpopulation of intermediate cells. (PPTX) [file pone.0211583.s001.pptx]

## Slide 1
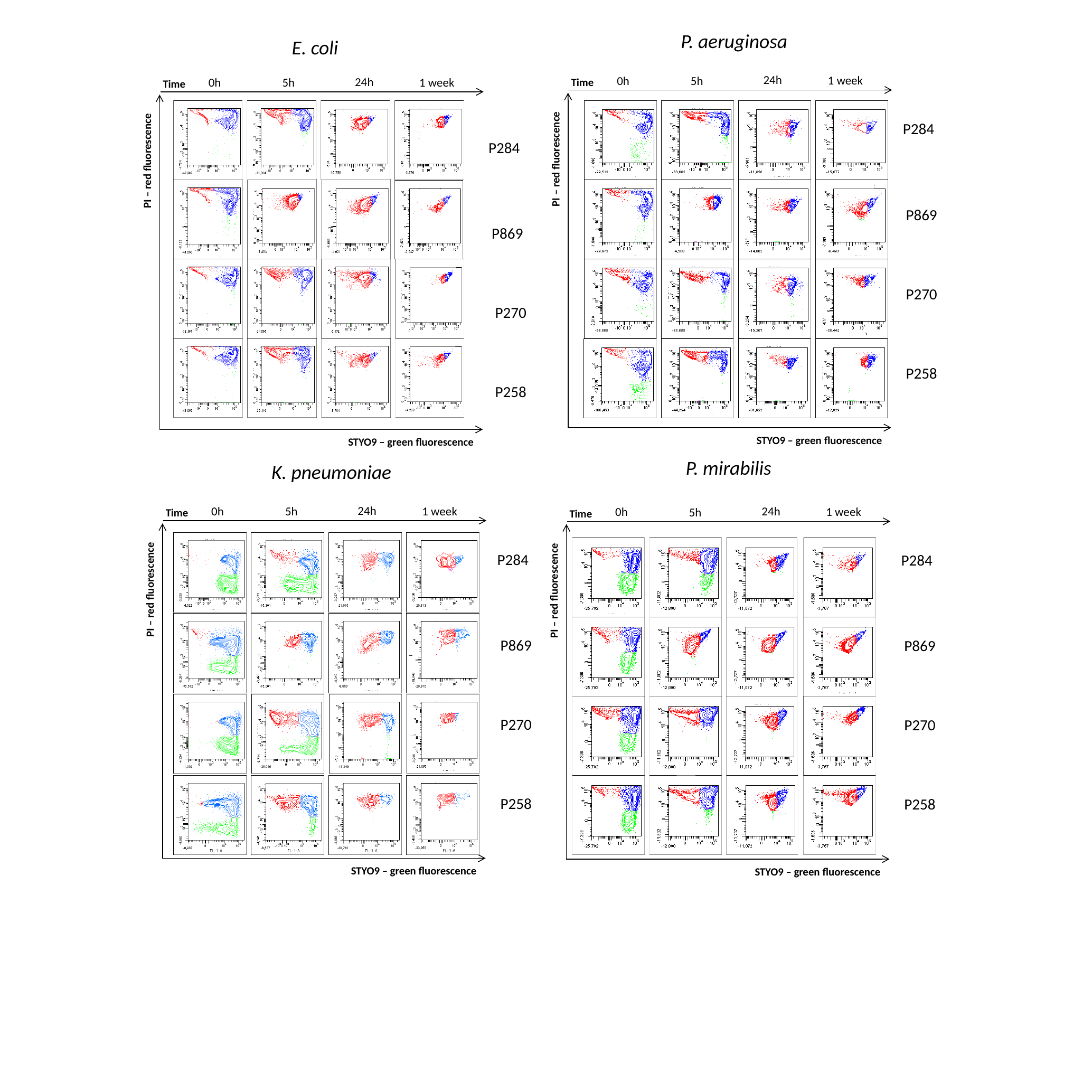

P. aeruginosa
24h
 1 week
0h
5h
Time
PI – red fluorescence
STYO9 – green fluorescence
 P284
P869
P270
P258
E. coli
24h
 1 week
0h
5h
Time
PI – red fluorescence
STYO9 – green fluorescence
 P284
P869
 P270
 P258
P. mirabilis
24h
 1 week
0h
5h
Time
PI – red fluorescence
STYO9 – green fluorescence
 P284
P869
P270
P258
K. pneumoniae
24h
 1 week
0h
5h
Time
PI – red fluorescence
STYO9 – green fluorescence
 P284
P869
P270
P258
